# Sugary beverage consumption mediates the relationship between late chronotype, sleep duration, and weight increase among undergraduates: a cross-sectional study

**DOI:** 10.1186/s12199-018-0754-8

**Published:** 2018-12-22

**Authors:** Wei Li, Mengyun Wu, Fang Yuan, Huadong Zhang

**Affiliations:** 1 Department of Occupational Health Surveillance, Chongqing, 400016 China; 2 Center for Disease Control and Prevention, Chongqing, 400016 China; 3 Chongqing Engineering research Center of Stem Cell Therapy, The Children’s Hospital of Chongqing Medical University, Chongqing, 400014 China

**Keywords:** Sleep duration, Chronotype, BMI, Sugary beverage, College student

## Abstract

**Background:**

The study aimed to explore whether sugary beverage consumption is a key mediator of late chronotype, sleep duration, and weight increase in college students in China.

**Methods:**

The cross-sectional study was conducted in four universities using a sample of 800 undergraduate students recruited by cluster sampling. A self-reported questionnaire was given out to collect information, including the characteristic of social demography, state of physical exercise and sugary beverage intake, the types of chronotype, and psychological conditions. Then, two structural equation models (SEMs) were constructed to test the mediating effect of sugary beverage consumption.

**Results:**

The significant indirect effect of sugary beverage consumption was found between chronotype and BMI (effect = − 0.03, SE = 0.01, 95% CI [− 0.05, − 0.02]) and between sleep duration and BMI (effect = − 0.12, SE = 0.05, 95% CI [− 0.16, − 0.09]). In addition, physical exercise and psychological condition also play mediating effects between chronotype and BMI (effect = − 0.04, SE = 0.01, 95% CI [− 0.06, − 0.01] and effect = − 0.03, SE = 0.02, 95% CI [− 0.05, − 0.01]), but their mediating effect was not found between sleep duration and BMI.

**Conclusion:**

Preventive measures should be taken to decrease student’s sugary beverage consumption, improve physical exercise, and increase individual well-being to avoid the harmful effects of eveningness. Moreover, the issue of short sleep duration among college students may be further investigated in future research.

## Introduction

Obesity has significantly become a severe public health problem, which has risen as a common social and medical phenomenon and is associated with the growth of the risk for chronic diseases, such as cancers, hypertension, type 2 diabetes, and cardiovascular disease [1–3]. Along with China’s increasing productivity level and people’s rapid consumption level over the past years, this issue has slowly attracted attention on overnutrition among the Chinese population [4, 5]. A cross-sectional survey found that China has encountered a severe prevalence of overweight/obesity at 45% in 2011 compared with 25% in 1991 [6–9]. Thus, exploring the epidemiological paths of obesity is extremely urgent to further conduct relevant control and prevention work.

In recent years, the relationship between sleep and weight has been mentioned [10–12]. It includes several factors, especially for sleep duration. Moreover, inadequate sleep duration may be associated with BMI increase [13]. However, few studies examined the causal relationship between behavioral risk (high-energy intake and low physical exercise), sleep factors, and weight outcome (e.g., overweight/obesity). In addition, chronotype as a characteristic sleep index has been overlooked on obesity research, and chronotype can obviously reflect individual sleep and physical statuses on the time dimension [14–17]. To illustrate, a cross-sectional survey of a large sample by Roeeneberg and team members found an inverse relationship between late chronotype and overweight/obesity [18]. Interestingly, chronotype slowly changed during adolescence and even early adulthood [19]. For example, departing from parental supervision and lacking a reasonable schedule, college students are inclined to late chronotype [20]. Moreover, individual’s socialization has also been reported as follows: college students preferred to seek social support, thereby triggering their willingness to join inter-recreational activities, which may lead to the development of a late chronotype [21]. Several college students also experience deleterious health behaviors, thereby rendering them vulnerable to weight gain and even overweight/obesity. Overweight/obese people would suffer from mental disorders, such as depression [22]. Baron and colleagues found that 12% of college students reported experiencing chronic depressive symptoms following sleep disturbance [23]. With respect to the relationship between eating patterns and chronotype, the individuals categorized as evening types consume a large amount of high-energy food and beverages than morning types and neutral types [24, 25]. This phenomenon might be because late chronotype prolongs individual drop asleep time, and these people feel more tired and hungry than the other types. Lack of sleep was correlated with a high-sugary beverage consumption and low physical exercise time [26, 27]. Notably, the issue of sugary beverage consumption pattern in China raises widespread concerns, although sugary beverages have been harmful associations with a higher incidence of type 2 diabetes, obesity, and cardiovascular [28, 29]. However, they are still popular in China such as Coca-Cola and PepsiCo carbonated drinks, and their sales drastically increased 149 and 129% in China, respectively [30]. Moreover, Geo et al. used the information from Chinese Nutrition and Health Surveillance: 2010–2012 (CHNS:2010–2012) to investigate the consumption of sugar-sweetened beverages among 18 years old and above. The longitudinal investigation involved 45,203 respondents and 150 sites of 31 provinces, which found Chinese adults consuming sugary beverages were 49.2% (men) and 50.8% (women), and carbonated beverages accounted for the highest proportion in beverages consumption (39.8%). The founding suggested that the government should develop nutritional education to reduce the consumption of sugary beverage [31]. University stage is a critical period for students to culture correct values and behavior habits. Because college students were first to leave the parents and obtain more responsible for arranging own schedule, some potential problems may emerge such as stimulation intake, and sugary beverages would become readily available products used widely. The abovementioned risk factors could provide a transitive path between sleep status and BMI. Overall, college students stand as the ideal population to explore the association between chronotype and BMI.

Descriptive studies on the association between sleep and BMI demonstrated that their correlation was identified by conducting univariate analysis and multiple linear regression. However, the findings were limited to direct effect of the factors and did not take interaction into consideration. Furthermore, sugary beverages’ use has the characteristics of behavior-related sleep and closely relates BMI, but the indirect effect role of sugary beverages on relationships between BMI, chronotype, and other behavior-related factors is still unclear. The structural equation modeling (SEM) analysis is the method considering the indirect effect of the variables, but few studies employed it in examining the effects of mediation on BMI. Therefore, to fill the above gaps in the current studies, we conducted two multiple mediation models to investigate the inter-effect relationship among chronotype, sleep duration, and BMI values, with sugary beverage and physical exercise time as mediators. The hypotheses were as follows: (1) chronotype, sugary beverage consumption, physical exercise status, and sleep duration have a direct effect on BMI; (2) sugary beverage consumption and physical exercise play a mediating role among chronotype, sleep duration, and BMI. This study will help support evidence to enhance prevention and intervention measures in promoting individual weight regulation among the college student population.

## Materials and methods

### Study design and sample

A total of 800 college students aged 19.8 ± 1.1 were recruited to participate in this study from four universities from April to July 2018 in Shapingba District, namely, Chongqing University, Chongqing Normal University, Southwest Politics and Law University, and Chongqing Foreign Language University. Twelve classes were randomly drawn out from the above universities (three classes for each university and ranging from freshmen to juniors). Then, all selected students were invited to complete this project. Self-reported questionnaires were distributed with students by their respective class monitors or teachers, and participants filled out the questionnaires for each question on the spot. Any problem during the investigation was explained by trained investigators, and informed consent was provided before questionnaire distribution. Information data were recorded by two investigators. Finally, we collected 788 completed questionnaires, with a response rate of 98.5%. Twelve questionnaires, which had many incomplete answers, were excluded. We included 788 respondent information in the last data processing and analysis.

### Primary measures

#### Demographic variable evaluation

All respondents’ socio-demographic information was collected by certain subjective items, including years, grade (grade 1/grade 2/grade 3), gender (female/male), height (cm) and weight (kg), residence (dormitory/apartment/home), types of university (Chongqing University/Chongqing, Normal University/Southwest Politics, and Law University/Chongqing Foreign Language University). BMI was calculated by weight (kg)/height (cm^2^) as continuous variable to fit mediation model. Furthermore, BMI was divided into four categories to present more basic information in descriptive analysis, namely, underweight (BMI < 18.5 kg/m^2^), normal weight (18.5 kg/m^2^ ≤ BMI < 24 kg/m^2^), overweight (24 kg/m^2^ ≤ BMI < 28 kg/m^2^), and obese (BMI ≥ 28 kg/m^2^).

#### Sleep duration and chronotype measures

Individual sleep pattern may show a significant difference between working days and rest days. Moreover, students could experience an inadequate sleep duration, even developing “sleep debt” during working days, and they would correct lost duration by prolonging the wake-up time during their free days. Therefore, mean sleep duration was measured as [(work day sleep duration time × 5) + (rest day sleep duration time× 2)]/7 over past week. We asked the participants to report their sleep onset time and sleep offset time; the night sleep duration values are calculated as (sleep offset time − sleep onset time). The individual chronotype was obtained using the Morningness-Eveningness Questionnaire (MEQ) [32], which comprised 19 questions associated with respondent’s individual sleep pattern according to their feeling during the latest several weeks, such as morningness, intermediate, and eveningness, as a valid tool determining the circadian typology in groups and reflecting the appropriate scale property in the Chinese population [33]. The MEQ score distributions range from 16 to 86. The higher total scores mean that the persons are classified as morning types, and the lower total scores show strong evening types. The morningness scores range from 59 to 86, the intermediate scores range from 42 to 58, and the eveningness scores range from 16 to 41. Cronbach’s *α* was 0.94 in this study.

#### Sugary beverage consumption and physical exercise time calculation

Sugary beverage consumption was assessed using the subjective problems: “How many bottles or tins of sugary beverage do you usually drinks on average per day over past week? (work days and rest days, respectively)” mean sugary beverage consumption was measured as [(work day consumption ×  5) + (rest day consumption × 2)]/7. Sugary beverages were defined as carbonated beverages, soft beverages, sweetened juices, and others. Then, reference standards were provided to participants that one bottle approximately equal to 500 ml and one tin approximately equal to 330 ml. Moreover, physical exercise time was also collected by an open question: “How much time for the past week do you usually spend on moderate exercise, such as carrying packet on average per day over past week (minutes/hours)? (work days and rest days, respectively)” mean physical exercise time was measured as [(work day physical exercise time × 5) + (rest day physical exercise time × 2)]/7. Moderate exercises include fast walking, dancing, doing homework, carrying medium weight goods, and so on.

#### Mental condition determination

The Depression Anxiety Stress Scale 21 (DASS-21) is a self-reported instrument with three subscales to diagnose individual psychological distress over past week, including anxiety, depression, and stress dimension [34]. Three subscales were classified into three levels, namely, low, moderate, and severe. Low scores were cut off as depression (≤ 13), anxiety (≤ 9), and stress (≤ 14); middle scores were cutoff as depression (14–20), anxiety (10–14), and stress (14–25); severe scores were cut off as depression (≥ 21), anxiety (≥ 15), and stress (≥ 26). The DASS-21 has been extensively applied for the Chinese students’ mental state study [35]. Cronbach’s α were 0.78, 0.82, and 0.84 in this study.

### Statistical analysis

SPSS version 22 was conducted for this statistical analyses (SPSS Inc., Chicago, IL, USA), and structural equation models were performed with SPSS PROCESS. Survey data were logged in Excel and checked by two people. Missing data were disposed using multiple imputations by the SPSS, and gender, grade, residence, and type of university were regarded as auxiliary variables to impute missing data, and the added times were 20. The descriptive analyses were employed to summarize the basic characteristics of data, which were shown as mean ± standard deviation (mean ± SD) or numbers and proportions (*N*, %). In addition, Pearson correlation analysis was operated to explore the possible relationship among independent variables, mediator, control variables, and dependent variables. Then, two mediation models were constructed to identify the direct/indirect effect of sugary beverage consumption. (1) The first model was set up among sleep duration, sugary beverage consumption, and BMI. (2) The second model was established among chronotype, sugary beverage consumption, and BMI. (3) To avoid exaggerating the influence of sugary beverage consumption, physical exercise time and metal state were brought into the two multiple mediation models. (4) To realize that the general data were no different among individuals in baselines, three control variables (gender, years, grade, residence) were selected in SEM. In this study, bootstrapping technique was used to estimate 10,000 resamples to reduce type I error [36, 37]. Indirect and direct effects were represented by bias-corrected 95% confidence intervals (95% CIs). The significant differences among all analyses were regarded as *p* < 0.05 and adopted the two-sided test.

## Results

### Characteristic of sample

All the charateristic are showed in Table 1. This study involved 800 undergraduate students. A total of 788 participants aged 19.8 ± 1.1, which include 271 (34.4) males and 517 (65.6%) females, completed all the questions. A total of 722 (91.6%) lived on campus, 26 (3.3%) lived in apartments, and 40 (5.1%) lived at home. A total of 212 (26.9%) were grade 1 students, 255 (32.4%) were grade 2 students, and 321 (40.7%) were grade 3 students. A total of 199 (25.3%) were from Chongqing University, 207 (26.3%) were from Chongqing Normal University, 174 (22.1%) were from Southwest Politics and Law University, and 208 (26.4%) were from Chongqing Foreign Language University. A total of 158 (20.1%) were underweight, 585 (74.2%) were normal weight, 32 (4.1%) were overweight, and 13 (1.6%) were obese. The average sleep duration was 7.6 ± 1.1 h, and the average physical exercise time was 4.6 ± 2.9 h. The average sugary beverage consumption was 0.7 ± 0.5 l. With regard to chronotype and mental state, a total of 172 (21.8%) were morningness, 495 (62.8) were intermediate, and 121 (15.4%) were eveningness. A total of 81 (10.2%) were middle/high depression, 521 (66.0%) were middle/high stress, and 161 (20.5%) were middle/high anxiety.

**Table 1:** Demographic characteristics of undergraduate students in Chongqing, China, 2018 (*n* = 788, response rate: 98.5%)

Variable	Number	Percentage (mean ± SD)
Age	788	19.8 ± 1.1
Grade		
Grade 1	212	26.9%
Grade 2	255	32.4%
Grade 3	321	40.7%
Gender		
Male	271	34.4%
Female	517	65.6%
Residence		
School	722	91.6%
Apartments	26	3.3%
Home	40	5.1%
Type of university		
Chongqing University	199	25.3%
Chongqing Normal University	207	26.3%
Southwest Politics and Law University	174	22.1%
Chongqing Foreign Language University	208	26.4%
BMI value		
BMI < 18.5(underweight)	158	20.1%
18.5 ≤ BMI < 24(normal weight)	585	74.2%
24 ≤ BMI < 28 (overweight)	32	4.1%
BMI ≥ 28(obesity)	13	1.6%
Sleep duration (hours)	788	7.6 ± 1.1
Chronotype		
Morningness	172	21.8%
Intermediate	495	62.8%
Eveningness	121	15.4%
Sugary beverage intake daily (L/day)	788	0.7 ± 0.5
Physical exercise time (hours)	788	4.6 ± 2.9
DASS-21		
Depression		
Low	707	89.7%
Middle	77	9.7%
High	4	0.5%
Stress		
Low	267	33.8%
Middle	514	65.2%
High	7	0.8%
Anxiety		
Low	627	79.6%
Middle	151	19.2%
High	10	1.3%

### Pearson correlation of main variables

The Pearson correlation analysis was conducted between continuous variables shown in Table 2. The significant positive correlation was found between age and BMI, age and sleep duration, BMI and sugary beverage intake, BMI and sleep duration, BMI and chronotype, sugary beverage consumption and DASS-21 scores, physical exercise time and chronotype, and BMI and DASS-21 scores. The significant negative correlation was found between BMI and physical exercise time, DASS-21 scores and chronotype, sugary beverage intake and sleep duration, and sugary beverage intake and chronotype.

**Table 2:** Correlation of main variables

	1	2	3	4	5	6	7
1. Age	1						
2. BMI	0.07*	1					
3. Sugary beverage intake	− 0.04	0.29 **	1				
4. Physical exercise time	− 0.05	− 0.12**	0.06	1			
5. Sleep duration	0.10**	0.19 **	− 0.12 **	− 0.04	1		
6. DASS-21	− 0.08*	0.28**	0.54 **	0.02	− 0.05	1	
7. Chronotype	− 0.04	0.51 **	− 0.15**	0.39**	− 0.11**	− 0.12**	1

*Note.* *p < 0.05; **p < 0.01

### Mediating role of sugary beverage use in the relationship between chronotype and BMI

**Fig. 1:**
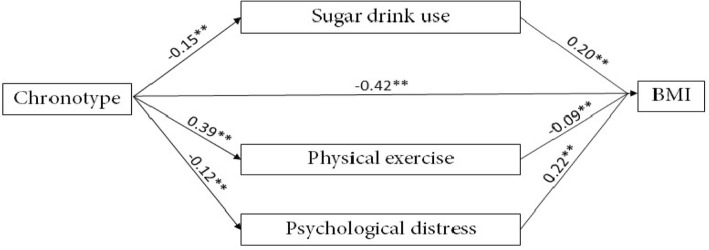
(Wei Li and Mengyun Wu). Mediating role of sugary beverage use in the relationship between chronotype and BMI. *Note.* ^**^p < 0.01

Figure 1 and Table 3 present the indirect and direct effects of the first mediation model. With regard to the effects of chronotype, the positive direct effects were found between chronotype and physical exercise time (*B* = 0.39, SE = 0.03, *p* < 0.01), and negative direct effects were found between chronotype and DASS-21 score (*B* = − 0.12, SE = 0.04, *p* < 0.01), chronotype and sugary beverage consumption (*B* = − 0.15, SE = 0.03, *p* < 0.01), and chronotype and BMI (*B* = − 0.42, SE = 0.04, *p* < 0.01). Therefore, a low chronotype score may generate an increased sugary beverage consumption, less physical exercise, and severe mental condition, even obesity.

**Table 3:** Direct mediating of role sugary beverage use in the relationship between chronotype and BMI

Paths	*B*	SE	95% CI	*p*
Chronotype				
BMI	− 0.42	0.04	(− 0.48, − 0.34)	< 0.01
Sugar drink use	− 0.15	0.03	(− 0.21, − 0.10)	< 0.01
Physical exercise	0.39	0.03	(0.33, 0.45)	< 0.01
Psychological distress	− 0.12	0.04	(− 0.20, − 0.05)	< 0.01
Sugar drink use				
BMI	0.20	0.02	(0.16, 0.25)	< 0.01
Physical exercise				
BMI	− 0.09	0.03	(− 0.15, − 0.03)	< 0.01
Psychological distress				
BMI	0.22	0.04	(0.15, 0.29)	< 0.01

*Note. SE* stand error, *95% CI* 95% confidence interval

The indirect effects of mediators were further checked, including sugary beverage intake, physical exercise time, and DASS-21 score. Significant indirect effects were found in the first mediation model, respectively, sugary beverage consumption (effect = − 0.03, SE = 0.01, 95% CI [− 0.05, − 0.02]), physical exercise time (effect = − 0.04, SE = 0.01, 95% CI [− 0.06, − 0.01]), and DASS-21 score (effect = − 0.03, SE = 0.02, 95% CI [− 0.05, − 0.01]). Moreover, the indirect effect of sugary beverage consumption and DASS-21 on BMI has a significant positive association (*B* = 0.20, SE = 0.02, *p* < 0.01 vs. *B* = 0.22, SE = 0.04, *p* < 0.01), thereby indicating that sugary beverage intake may be an undesirable factor between late chronotype and high BMI. In addition, the indirect effect of physical exercise time on BMI shows a significant negative association (*B* = − 0.09, SE = 0.03, *p* < 0.05). Thus, physical exercise time can be considered a protective factor between late chronotype and high weight. The total effect model has contributed approximately 26% of the variables in BMI (*R*^2^ = 0.26, *p* < 0.01). Table 4 reports the indirect mediation of sugary beverage use in the model.

**Table 4:** Indirect mediating role of sugary beverage use in the relationship between chronotype and BMI

Domain	Effect (boot SE)	Lower, upper 95% confidence interval
Sugary beverages intake	*− 0.03(0.01)*	*(− 0.05,-0.02)*
Physical exercise time	*− 0.04(0.01)*	*(− 0.06,-0.01)*
DASS-21 scores	*− 0.03(0.02)*	*(− 0.05,-0.01)*

*Note.* Significant mediation is highlighted in italic font

### Mediating role of sugary beverage use in the relationship between sleep duration and BMI

**Fig. 2:**
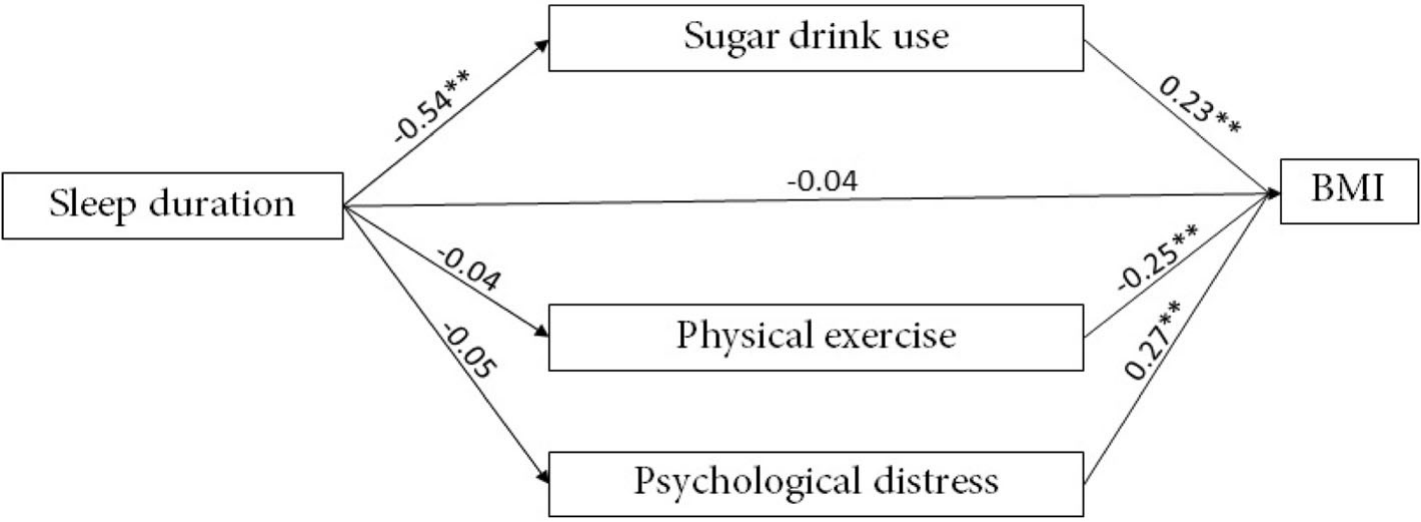
(Wei Li and Mengyun Wu). Mediating role of sugary beverage use in the relationship between sleep duration and BMI. Note.^**^p < 0.01

Figure 2 and Table 5 present the indirect and direct effects of the first mediation model. Concerning the effects of sleep duration, the positive direct effects were found between sleep duration and sugary beverage intake (*B* = − 0.54, SE = 0.03, *p* < 0.01), sleep duration, and BMI (*B* = − 0.13, SE = 0.03, *p* < 0.01). Thus, these results may point out that insufficient sleep duration would aggravate the risk of overweight/obesity and additional sugary beverage intake. However, the significant association was not found between sleep duration and physical exercise time and between sleep duration and the DASS-21 scores.

**Table 5:** Direct mediating role of sugary beverage use in the relationship between sleep duration and BMI

Paths	*B*	SE	95% CI	*p*
Sleep duration				
BMI	− 0.04	0.03	(− 0.02, 0.10)	0.22
Sugar drink use	0.54	0.03	(0.48, 0.59)	< 0.01
Physical exercise	− 0.04	0.04	(− 0.11, 0.03)	0.25
Psychological distress	− 0.05	0.04	(− 0.13, 0.04)	0.26
Sugar drink use				
BMI	0.23	0.03	(0.17, 0.28)	< 0.01
Physical exercise				
BMI	− 0.25	0.03	(− 0.30, − 0.19)	< 0.01
Psychological distress				
BMI	0.27	0.04	(0.19, 0.36)	< 0.01

*Note*. *SE* stand error, *95% CI* 95% confidence interval

The indirect effect of mediators was explored by bootstrapping resampling. Interestingly, the significant indirect effects of sugary beverage intake were also found in the model (effect = − 0.12, SE = 0.05, 95% CI [− 0.16, − 0.09]), and the direct effect of sugary beverage intake on BMI was positive (*B* = 0.23, SE = 0.03, *p* < 0.05). Therefore, sugary beverage consumption may also be a risk factor on the association between sleep duration and BMI. The total effect model has contributed approximately 21% of the variables in BMI (*R*^2^ = 0.21, *p* < 0.01). Table 6 reports the indirect mediation of sugary beverage use in the model.

**Table 6:** Indirect mediating role of sugary beverage use in the relationship between sleep duration and BMI

Domain	Effect (boot SE)	Lower, upper 95% confidence interval
Sugary beverages intake	*− 0.12(0.05)*	*(− 0.16,-0.09)*
Physical exercise time	0.01(0.01)	(− 0.01,0.03)
DASS-21 scores	− 0.01(0.01)	(− 0.04, 0.01)

*Note.* Significant mediation is highlighted in italic font

## Discussion

Chronotype and sleep duration are often associated with human weight regulation [18, 20, 38]. In addition, daily behavior habits that may not be neglected are attached to the individual sleep pattern, such as sugary beverage intake and physical exercise. In this study, we attempted to explore that sugary beverage intake significantly mediates cross-sectional associations between sleep duration, chronotype, and BMI; moreover, the significant indirect effect of sugary beverage intake existed in the two models. These novel results may enrich the relationship of the paths between sleep and weight, suggesting that good sleep habits may promote a fit weight and that this process may be developed through low-sugary beverage intake.

In this study, the mediating role of sugary beverage intake was illustrated in the two models, and these results were supported by previous studies [39, 40]. Late chronotype and short sleep duration may be associated with high-sugary beverage intake among adolescents and students. To illustrate, Sampasa-Kanyinga and colleagues conducted a cross-sectional study, including 9473 adolescents aged 11–20 years old, which reported that short sleep duration was associated with 1.64 odds of sugary beverage consumption in middle school students [39]. Moreover, Kanerva and colleagues examined 4493 college subjects’ socio-demographic backgrounds, sleep patterns, and dietary intake habits. Using these data, they found that subjects in the lowest chronotype scores consumed more soft beverages than the highest group [40]. The results of our study extend previous findings and add to our views and opinions by suggesting that chronotype and sleep duration were associated with BMI, and this relationship was mediated by sugary beverage intake.

The potential mechanism on sleep pattern and sugary beverage consumption was not thoroughly explained, probably for several reasons. First, the individual with a late chronotype or short sleep duration may experience an irregular eating habit and a perceived hunger, even overeating [41]. A meta-analysis by Cappuccio demonstrated that the pooled odds ratio for short sleep duration and obesity was 1.89 in children and 1.55 in adults [42]. These outcomes may intensely relate to hormonal secretion in the body. Inefficient sleep may influence hormone levels, such as satiety hormones, leptin, and ghrelin [43, 44]. Epidemiological studies have also demonstrated that late chronotype or short sleep duration was associated with significantly high insulin and low leptin levels in the college population [20, 45]. Second, late chronotype and short sleep duration would generate sleep debt, but students would go to school in the morning the next day, and the risk of excessive daytime sleepiness would increase; then, sugary beverage consumption would play a stimulant role in improving individual attention [39, 46]. Third, late chronotype and short sleep duration could develop into sleep disorders in severe condition, and this condition means that an individual’s immune function will decrease and levels of inflammation increase. Inflammation regulates energy metabolism in both physical and mental conditions. An individual’s chronic inflammatory response, when not effectively controlled, leads to energy metabolism disorders and insulin resistance [47]. Hence, patients’ outcome would deteriorate if they consume sugary beverages.

Physical exercise as significant to maintaining the stabilization of the human body is common knowledge, including physical and psychological health [48]. The association between sedentary behavior and other physical inactivity and cardiovascular disease, type 2 diabetes, and obesity have attracted significant attention in the field of public health [49]. In our study, the significant indirect effect of physical exercise time was found between chronotype and BMI. A systematic review generated similar results, which found that morningness could have a merit in perceived exertion, less fatigue, and better performance in submaximal physical exercise and athletic tasks [50]. One reasonable explanation is that the individual physical exercise state is influenced by exogenous and endogenous factors, such as exercising at day or night time and when to awake, and eveningness seemingly requires an extra time for physical preparations after getting up. However, the indirect effect was not found between sleep duration and BMI. This reason may be attributed to the differences in measurement methods: the sleep duration was calculated by individual average sleep time, which has definite limitations. If one person has a sleep onset at 21:00 and sleep offset 9:00, he/she has slept for 12 h, but if he/she goes to bed at 24:00 and gets up at 12:00, he/she has also slept for 12 h. In other words, average sleep duration only counts for your total sleep time rather than when you go to sleep. By contrast, chronotype was calculated not only by inquiring on sleep time but also by asking one’s sleep habit and physical condition. Hence, late chronotype refers to individuals who go to bed late and get up late; thus, they may lose considerable time for exercise and activities. Moreover, daytime napping would happen frequently in eveningness, leading the person to feel tired and less inclined to go outside.

Finally, subjects’ psychological conditions were evaluated in the two models. The significant indirect effects were found in the first model but not in the second model. Previous studies showed that late chronotype could also impair individual sleep quality and increase dietary intake owing to psychological factors, such as depression and stress [51, 52]. Rique and colleagues found that eveningness was associated with poor quality of sleep among medical students, which may be explained by the medical college students’ extra academic pressure and thus compelled them to shorten sleep time [51]. Another study (Romo-Nava et al.) also found that eveningness increased the susceptibility for depression than morningness and intermediate; moreover, late chronotype and depression were both associated with severe perceived academic stress [52]. This conclusion further indicated the diverse effect of excessive study burden for student’s sleep habits. Interestingly, we found no mediation effect of mental condition in the second model. Previous research has elaborated the association between sleep duration and depression, and their relationship may be found using the U-shape graph [53], suggesting that the individual who experienced increased or decreased sleep duration may likely lead to a depressive symptom. Two reasons explain the inconsistency of our study with the above literature. First, the mediating effect of sugary beverage intake was strong in the model and weakens the indirect effect of mental conditions. Moreover, an increased stimulant intake, such as alcohol, coffee, and carbonated beverages may aggravate depression, and the inter-effect of stimulant intake and depression requires further investigation in longitudinal studies [54]. Second, most results of participants’ DASS-21 scores were mostly concentrated in the middle and low levels with a skewness distribution. Hence, a large sample survey should be conducted to achieve comprehensive psychological conditions in college students.

This study has definite limitations. First, the method design adopted a cross-sectional survey, and information and date cannot determine the results of causality. Second, the ratio of female is apparently higher than that of male in the survey population, and males and females may have significant differences in routine life. Thus, the sex bias should be focused in this study. Third, the self-reported questionnaires were used to collect information on physical exercise time and sugar drink consumption, which has the advantages of simplicity, low cost, high response rate, and convenience in processing numerical results. However, this method was hard to achieve precise quantification and lacked of some reliabilities and validities. This would be an important limitation of our study. Hence, objective measures should be adopted in future nutritional epidemiology research, such as food logs and actigraphy measured exercise parameters. Furthermore, we should pay more attention to the practical application and let the respondents know the meaning of questionnaire and minimize experimental errors. Fourth, the research was restricted to Chongqing, and individual chronotype may have an impact by living at what latitude. Thus, the result may lack richness and extensibility. Fifth, although we used the bootstrapping technique to decrease the type I error and remedy the inferiority of the sample, the large sample survey could be further carried out to cover different students with diverse majors. Finally, the kind of sugary beverages was not distinguished in this study, and diverse kinds of stimulant beverages may generate different effect sizes in the model.

## Conclusions

This study found that sugary beverage intake might mediate the association among sleep duration, late chronotype, and weight gain among college students. These results could provide a possible new approach to explore the effect of food between sleep pattern and weight, which suggests that sugary beverage intake may be a risk factor to developing overweight/obesity for eveningness. In addition, the schools should take relevant measures to decrease frequency of this exposure. For example, schools could reduce students’ academic burdens after class and arrange similar chronotypes to live together. Moreover, the individual psychological conditions and physical activities have significant indirect effects between chronotype and BMI, which indicated that college students should develop a culture of effective exercise and nurture a good mind to decrease the negative effect of late chronotype on weight.

## Abbreviations

BMI: Body mass index
CI: Confidence interval
DASS-21: The Depression Anxiety Stress Scale 21
MEQ: The Morningness-Eveningness Questionnaire
SD: Standard deviation
SE: Standard error
SEM: Structural equation model
SPSS: Statistical Package for Social Science
